# Vascular Endothelial Integrity Affects the Severity of Enterovirus-Mediated Cardiomyopathy

**DOI:** 10.3390/ijms22063053

**Published:** 2021-03-17

**Authors:** Jin-Ho Park, Ha-Hyeon Shin, Hyun-Seung Rhyu, So-Hee Kim, Eun-Seok Jeon, Byung-Kwan Lim

**Affiliations:** 1 Department of Biomedical Science, Jungwon University, 85 Munmu-ro, Goesan-eup, Goesan-gun, Chungbuk 28024, Korea; pjh718g@gmail.com (J.-H.P.); hahyun925@naver.com (H.-H.S.); rhyuhs@jwu.ac.kr (H.-S.R.); sohee5404@naver.com (S.-H.K.); 2 Division of Cardiology, Samsung Medical Center, Sungkyunkwan University School of Medicine 50 Irwon dong, Gangnam-gu, Seoul 06351, Korea; eunseok.jeon@samsung.com

**Keywords:** CVB3, myocarditis, endothelial junction, vascular permeability

## Abstract

Coxsackievirus and adenovirus receptor (CAR) is present in epithelial and vascular endothelial cell junctions. We have previously shown a hemorrhagic phenotype in germ-line CAR knock-out mouse embryos; we have also found that CAR interacts with ZO-1 and β-catenin. However, the role of CAR in vascular endothelial junction permeability has not been proven. To understand the roles of CAR in the vascular endothelial junctions, we generated endothelium-specific CAR knockout (CAR-eKO) mice. In the absence of CAR, the endothelial cell layer showed an increase in transmembrane electrical resistance (TER, Ω) and coxsackievirus permeability. Evans blue dye and 70 kDa dextran-FITC were delivered by tail vein injection. We observed increased vascular permeability in the hearts of adult CAR-eKO mice compare with wild-type (WT) mice. There was a marked increase in monocyte and macrophage penetration into the peritoneal cavity caused by thioglycolate-induced peritonitis. We found that CAR ablation in endothelial cells was not significantly increased coxsackievirus B3 (CVB3) induced myocarditis in murine model. However, tissue virus titers were significantly higher in CAR-eKO mice compared with WT. Moreover, CVB3 was detected in the brain of CAR-eKO mice. Endothelial CAR deletion affects the expression of major endothelial junction proteins, such as cadherin and platelet endothelial cell adhesion molecule-1 (PECAM-1) in the cultured endothelial cells as well as liver vessel. We suggest that CAR expression is required for normal vascular permeability and endothelial tight junction homeostasis. Furthermore, CVB3 organ penetration and myocarditis severities were dependent on the endothelial CAR level.

## 1. Introduction

The endothelium is located on the inner side of all vessel types and is covered by a monolayer of endothelial cells. Endothelial cells lining the blood vessel wall are connected by adherens, tight, and gap junctions. These junctions are similar to epithelial junctions but with notable differences in terms of specific molecules and organization. Endothelial proteins play important roles in tissue integrity but also in vascular permeability, extravasation, and angiogenesis. For example, VE-cadherin is an endothelial cell-specific immunoglobulin-like protein localized in adherens junctions, where it interacts with structural proteins and signaling molecules such as β-catenin [1,2,3,4,5,6]. The null mutation of VE-cadherin prevents vascular remodeling and induces degeneration and regression of vascular structures, leading to early fetal demise [7,8]. Endothelial cell-restricted inactivation of the β-catenin gene also causes a defective vascular pattern and increases vascular fragility [9,10]. In addition, endothelial cell-selective adhesion molecule (ESAM) is a member of the immunoglobulin receptor family that also mediates homophilic interactions between endothelial cells. Targeted disruption of endothelial ESAM inhibits angiogenesis [11,12]. Therefore, adhesion molecules contribute to the organization of endothelial cells into vessels, angiogenesis, and the maintenance of vascular integrity and paracellular permeability.

In polarized epithelial cells, coxsackievirus and adenovirus receptor (CAR) is located at the basolateral surface and specifically concentrates at tight junctions, where it co-localizes with the tight-junction protein zonula occludens, ZO-1 [13,14]. CAR also co-localizes with adhesion proteins such as E-cadherin [15] and β-catenin [14]. It is also well known as a co-ligand for junction adhesion molecule like (JAML), which is a cell signaling receptor of the immune system implicated in asthma, cancer, and chronic nonhealing wounds [16]. The overexpression of CAR can lead to an increase in the electrical resistance of a cell monolayer [13]. In addition, CAR mediates homophilic interactions with CAR molecules on neighboring cells [13,17,18]. Therefore, CAR has been considered as a potential transmembrane component of adhesion and tight junctions in the endothelium layer. CAR plays an important role in coxsackievirus B3 (CVB3) related pathology and adenoviral gene therapy. CAR is a 46-kDa integral membrane protein that belongs to the immunoglobulin G superfamily of protein, similar to other adhesion molecules [19,20,21,22]. The tissue expression pattern of CAR varies significantly depending on the stage of embryonic development and tissue differentiation. For example, CAR is strongly expressed in the brain and heart of mouse embryo until the neonatal phase, but its expression decreases in adult mice [19]. Although the roles of CAR in embryonic development, cell adhesion, tumor cell growth, inflammation, and tissue regeneration have been reported, the underlying mechanisms remain elusive.

CAR has been found on endothelial cells of blood vessels [22] and is expressed in cultured human umbilical vein endothelial cells (HUVECs); its expression level in HUVECs increases in response to high cell density [20,21,22]. Previously, we reported a mouse knockout of CAR. The homozygous embryos died between E11.5 and E12.5, and approximately 50% of E10.5–11.5 CAR-null embryos presented vascular dilation and hemorrhage in vasculature-rich areas such as brain, heart, and the dorsal vessels and frequently showed pericardial effusion [23], suggesting that permeability of the microvasculature was increased in the absence of CAR. Together, the available data suggest that CAR in endothelial cells is required for vascular morphogenesis and maintenance of vascular integrity. 

In this study, we examined the role of CAR in the endothelium and elucidated its physiological function in vascular endothelial cells in maintaining the vessel integrity and paracellular permeability during coxsackievirus infection. This study could have important implications in the treatment of vascular diseases and eventually in understanding the role of CAR in viral heart diseases.

## 2. Results

### 2.1. Increase in CVB3 Permeability in Cultured Endothelial Cells 

**Figure 1:**
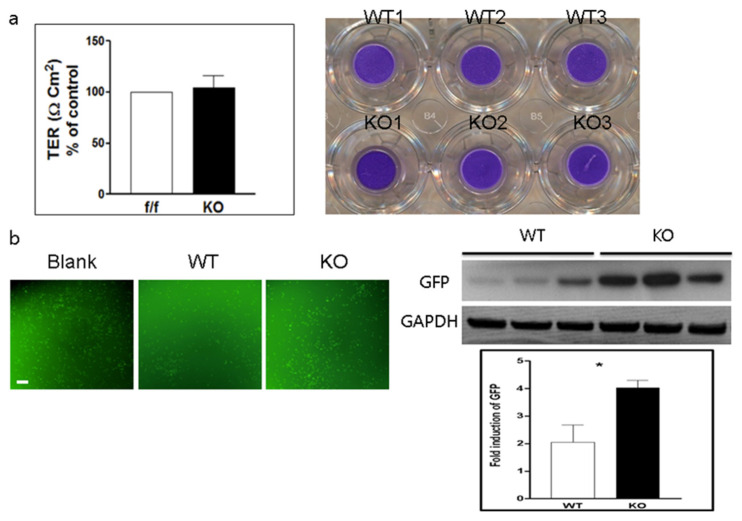
Endothelial coxsackievirus and adenovirus receptor (CAR) deletion increases permeability of endothelial cell monolayer for coxsackievirus B3 (CVB3). (**a**). Trans-epithelial electrical resistance (TER) was measured in a trans-well chamber with cultured wild-type (WT) endothelial cells and endothelial cells in which CAR had been deleted (CAR-eKO (KO)). Trypan blue staining showed endothelial cells in the upper well. (**b**). WT and KO endothelial cells were treated with Green fluorescent protein (GFP)-CVB3, and GFP expression was observed under a fluorescent microscope. Blank, no endothelial cell cultured chamber. (**c**). Cell extracts were subjected to Western blot analysis using the indicated antibodies. GFP immunoblot bands indicate fold changes of GFP normalized to glyceraldehyde 3-phosphate dehydrogenase (GAPDH) bands. All data are the mean ± s.e.m. from 3 independent experiments (scale bar, 100 µm). * *p* < 0.05 by two-tailed Student’s *t*-test.

To examine the effect of CAR deletion, isolated wild-type (WT) endothelial cells and endothelial cells from mice in which CAR had been deleted from the endothelial cells (CAR-eKO) (KO) were cultured in the upper chamber and HeLa cells were cultured in the bottom chamber of a trans-well plate (Nunc Co., UK). Green fluorescent protein-Coxsackievirus B3 (GFP-CVB3) [24] was added to the upper chamber and the plate was incubated for 18 h. Trans-epithelial resistance (TER) was measured to assess the transcellular permeability of endothelial cells; after TER measurement, the endothelial cells in the upper chamber were stained with crystal violet to assess cell density. TER was similar in WT and KO endothelial cells. There were no electrical resistance changes in endothelial cells due to CAR deletion, and crystal violet staining confirmed a similar cell density of WT and KO endothelial cells (Figure 1a). However, the GFP expression of bottom well HeLa cells was significantly increased in the CAR deleted endothelial cell cultured chamber compared to the WT endothelial cell cultured chamber, suggesting that the permeability of GFP-CVB3 was significantly increased in the CAR deleted endothelial cells, blank indicated no endothelial cell cultured chamber (Figure 1b). These results strongly imply that CAR deletion is closely related to vascular permeability for CVB3. 

### 2.2. Endothelial Cells Delay Virus Penetration

**Figure 2:**
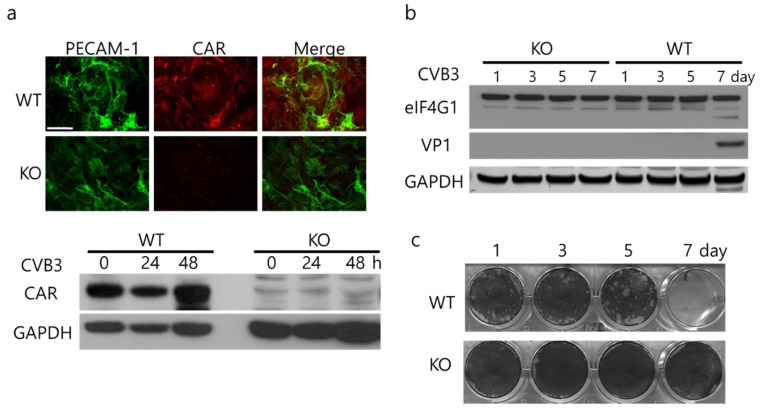
CVB3 replication is delayed in endothelial cells. (**a**). WT and CAR-eKO (KO) endothelial cells were immunostained with antibodies against platelet endothelial cell adhesion molecule-1 (PECAM-1, green) and CAR (red) and analyzed by fluorescence microscopy (scale bar, 40 µm). Cell extracts were subjected to Western blot analysis using indicated antibodies. (**b**). WT and KO endothelial cells were treated with 1 m.o.i. CVB3 and then the cells were lysed at day 1, 3, 5, 7. Endothelial cell extract proteins were subjected to Western blot analysis using indicated antibodies. (**c**). Supernatants of CVB3-infected WT and KO cells were subjected to plaque forming unit (PFU) assay to measure release of virus into the supernatant.

CAR expression and CVB3 infection were confirmed in cultured hepatic endothelial cells. Cell shape and culture patterning of CAR-deleted endothelial cells was not significantly different to WT endothelial cells. The KO cells did not form endothelial tubes and showed weak platelet endothelial cell adhesion molecule-1 (PECAM-1) expression (Figure 2a). In the endothelial cells, CVB3 replication was very slow. The seven days after CVB3 infection, viral capsid protein VP1 and progeny viruses were detected in WT endothelial cells but not in KO endothelial cells (Figure 2b,c). While WT-endothelial cells can be infected with CVB3, the infection occurs more slowly than in HeLa cells where there is usually robust replication and cell lysis by 24–48 h. Given this finding, the increase in passage of CVB3 in the trans-well chambers mentioned above indicate that the increase in CVB3 in the trans-well chambers that had CAR-eKO endothelial cells was most likely related to increased permeability of the endothelial cells to CVB3. 

### 2.3. Increased Vascular Permeability in the Heart of CAR-eKO Mice

**Figure 3:**
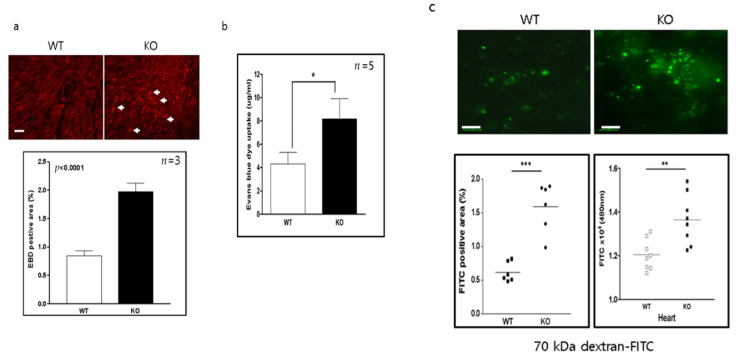
Vascular permeability is increased in the heart of endothelial CAR knockout mice. (**a**). WT and KO mice were injected with 10% Evans blue dye (EBD) for 3 h. Heart was sectioned and subjected to fluorescence microscopy to determine the area of EBD uptake (white arrows; scale bar, 100 µm). (**b**). EBD concentration in the heart extract was measured by microplate reader (*n* = 5 per group). (**c**). WT and KO mice were treated with 70 kDa dextran-FITC, and heart sections were examined by fluorescence microscopy to determine the area of FITC uptake (left). Dextran-FITC concentration in the heart extract was measured at 488 nm in a microplate reader (right) (*n* = 8 per group; scale bar, 5 µm). All data are the mean ± s.e.m. * *p* < 0.05, ** *p* < 0.01, and *** *p* < 0.001 by two-tailed Student’s *t*-test.

Evans blue dye (EBD) is used in some situations as a marker of vascular permeability. KO mice were assayed for vascular permeability by measuring the amount of Evans blue dye (EBD) uptake into the heart. Sixty minutes later the heart was removed, and the area and extent of EBD uptake was quantified (expressed as % of total). The EBD uptake was significantly increased in KO mice compared to WT mice (WT vs. KO, 0.9 ± 0.1 vs. 2 ± 0.1, respectively; *p* < 0.0001) (Figure 3a). The concentration of EBD was also significantly increased in the heart of KO mice (WT vs. KO, 4.1 ± 1.1 vs. 8 ± 1.6 μg/mL, *p* < 0.05) (Figure 3b). However, the EBD molecules were smaller than those of conventional biomaterials such as albumin, immunoglobulin, and viruses. To examine the vascular permeability of a larger substance, 75-kDa dextran (Dextran-FITC) was delivered through the tail vein, and its uptake levels in the heart were measured. The area of Dextran-FITC was significantly increased in the hearts of KO mice (WT vs. KO, 0.6 ± 0.2 vs. 1.59 ± 0.18 µg/mL, *p* < 0.001). The level of dextran-FITC was also significantly higher in heart extracts from KO mice than in those of WT mice (WT vs. KO, 1.2 ± 0.2 vs. 1.39 ± 0.16; *p* <0.01) (Figure 3c). These data indicate that endothelial CAR deletion increases vascular permeability in the heart.

### 2.4. Increased Inflammatory Cell Infiltration into the Peritoneal Cavity in CAR-eKO Mice

**Figure 4:**
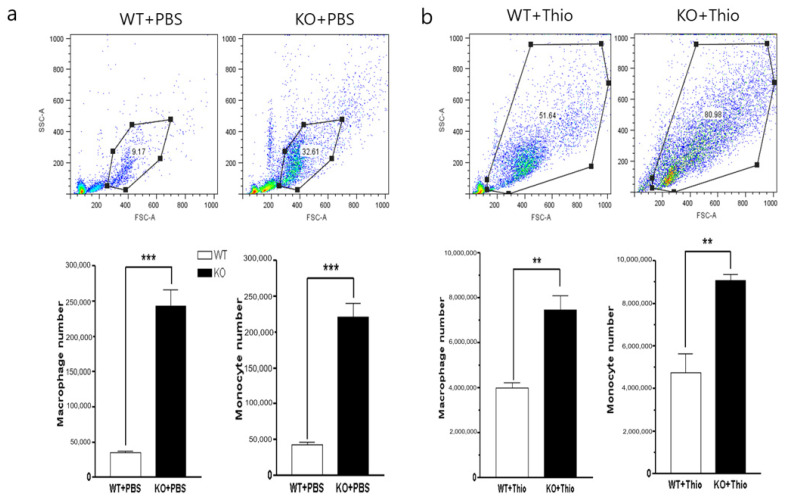
Endothelial CAR deletion increases inflammatory cell infiltration into the peritoneal cavity. (**a**,**b**). Ten-week-old male WT and KO mice were treated with PBS or 3% thioglycolate for 7 days, and peritoneal cells were isolated, stained with the indicated antibodies (monocytes = Gr-1 and macrophages = CD11b) and subjected to fluorescence-activated cell sorting (FACS) analysis. All data are the mean ± s.e.m. from 3 independent experiments. ** *p* < 0.01 and *** *p* < 0.001 by two-tailed Student’s *t*-test.

Vascular permeability affects the permeation of substances through blood vessels into the interstitial space of tissue. The migration of immune cells across the endothelium is closely related to the stability of endothelial cell tight junctions, and the direct infiltration of inflammatory immune cells will cause more tissue damage due to excessive inflammatory responses at the injured site. We observed the peritoneal cavity inflammatory cell infiltration with or without stimulation of inflammation. The baseline number of inflammatory cells into peritoneal cavity was measured in WT and CAR-eKO mice where cells were obtained by washing the peritoneum with PBS. We found that the total numbers of immune cells, macrophages (CD11b), and monocytes (Gr-1) that infiltrated into the peritoneal cavity were significantly higher in KO mice than in WT mice even without induction of inflammation (Figure 4a). The infiltration of inflammatory cells into the peritoneal cavity after induction of inflammation by thioglycolate injection was greatest in the KO mice. The number of peritoneal cells was significantly increased in WT and KO mice compared with untreated. The numbers of macrophages and monocytes were significantly greater in CAR-eKO mice than in WT mice (Figure 4b). These data suggest that endothelial CAR deletion increases inflammatory cell permeability and results in an increase in migration of monocytes and macrophages to the injured tissues.

### 2.5. Endothelial CAR Deletion Increases CVB3 Virus Titer without Changing the Extent of Inflammation 

**Figure 5:**
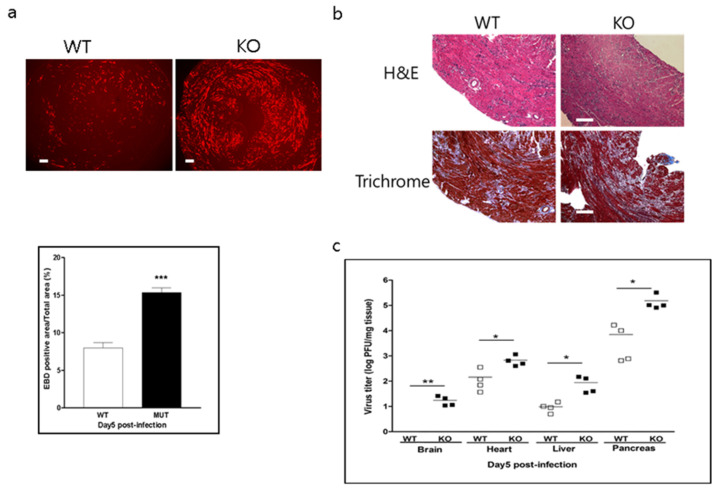
Effect of CAR deletion on CVB3-induced myocarditis. (**a**). Six-week-old male WT and KO mice were infected with 1 m.o.i. CVB3 for 5 days and then injected with EBD before being sacrificed. A heart section was examined by fluorescence microscopy. Red fluorescence represents EBD uptake by damaged myocardium (upper). The percent area of EBD was quantified with Scion Image (*n* = 4 per group). (**b**). Histological findings of sectioned heart show inflammatory cell infiltration and fibrosis using hematoxylin & eosin (H&E) and trichrome staining. (**c**). Virus titers in organs of WT and KO mice determined by PFU assay (*n* = 4 per group). All data are the mean ± s.e.m. from 3 independent experiments performed (scale bar, 100 µm). * *p* < 0.05, ** *p* < 0.01, and *** *p* < 0.001 by two-tailed Student’s *t*-test.

To examine the effect of endothelial CAR deletion on myocarditis, WT and CAR-eKO mice were infected with CVB3. Five days after infection, heart, liver, pancreas, and brain were harvested to assess histology and extent of virus proliferation within the tissue. In Figure 5a, EBD uptake into the damaged myocardium by CVB3 was significantly higher in CAR-eKO than in WT mice (WT vs. KO, 8.2 ± 0.5 vs. 15.1 ± 0.6; *p* < 0.001). Heart inflammation and fibrosis were not significantly increased in CAR-eKO mouse hearts at 5 days post infection (Figure 5b). Virus replication in each tissue was confirmed by plaque forming unit (PFU) assay; tissue virus titer dramatically increased in KO mice compared with WT mice (Figure 5c). Interestingly, the virus was also detected in the brain of KO mice. Therefore, the endothelial cell CAR might be related to normal function of the blood–brain barrier (BBB) and block virus penetration from blood vessels into the brain.

### 2.6. CAR Stabilizes Endothelial VE-Cadherin and PECAM-1

**Figure 6:**
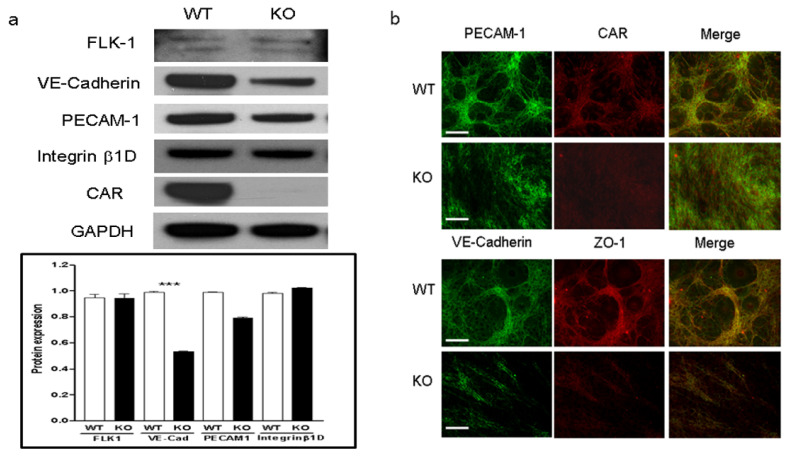
CAR deletion disrupts endothelial cell VE-cadherin and PECAM-1 expression. (**a**). Endothelial cells were isolated from the livers of WT and KO (with deleted CAR) mice. Cell extracts were subjected to Western blot analysis using the indicated antibodies. (**b**). Immunofluorescence staining of endothelial cells to determine changes in cell morphology. All data are the mean ± s.e.m. from 3 independent experiments (scale bar, 60 µm). *** *p* < 0.001 by two-tailed Student’s *t*-test.

To define the main causes of the abnormal vascular permeability in eCAR-KO mice, we examined the intrinsic changes in endothelial cells. In cultured hepatic endothelial cells, CAR deletion significantly decreased the expression of VE-cadherin and PECAM-1 in Western blot analysis, which regulate cell-to-cell permeability and functional activity of endothelial tight junctions (Figure 6a). Furthermore, the loss of VE-cadherin in endothelial cells resulted in loss of junction formation, normal cell-to-cell connection, and tube formation in immunofluorescent stain (Figure 6b). 

### 2.7. VE-Cadherin and PECAM-1 Expression in the Liver of KO Mice

**Figure 7:**
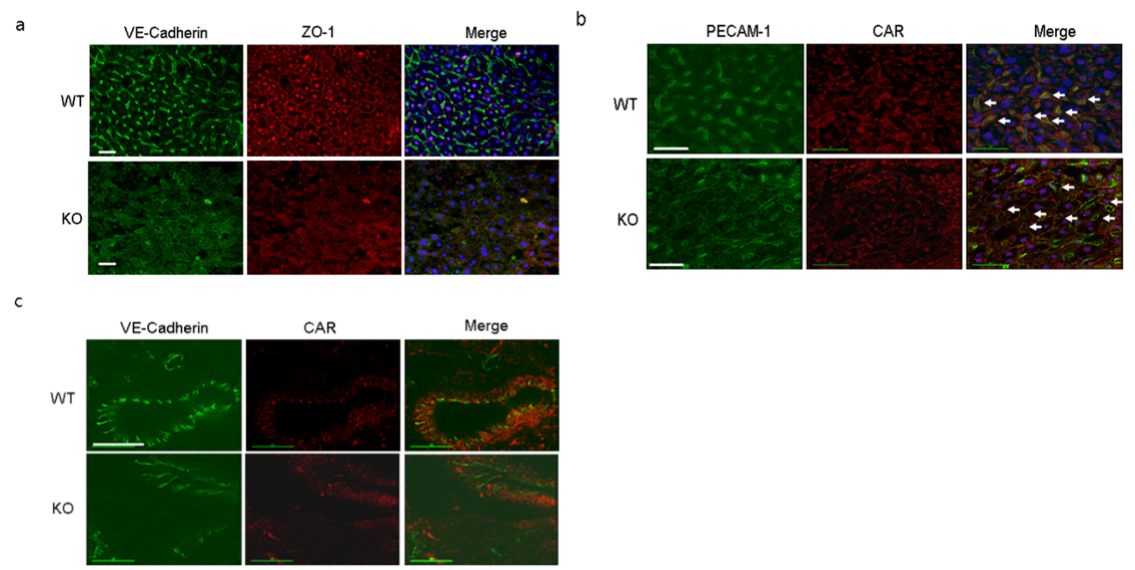
Disruption of the endothelial cell VE-cadherin and PECAM-1 expression by CAR ablation. (**a**). WT and KO mice liver were subjected to immunofluorescence staining using the indicated antibodies. Green, VE-cadherin and red, ZO-1. (**b**). Green, PECAM-1 and red, CAR in the vascular endothelial cells of liver, which are indicated by white arrows (scale bar, 40 µm and 15 µm). (**c**). WT and KO mouse hearts coronary vessel were subjected to immunofluorescence staining using the indicated antibodies. Green, VE-cadherin and red, CAR (scale bar, 15 µm).

Endothelial VE-cadherin and PECAM-1 are very important for maintaining vascular stability and permeability. Livers and hearts of 16-week-old WT and KO mice were immunostained with antibodies probed VE-cadherin, ZO-1, PECAM-1, and CAR. The levels of VE-cadherin and PECAM-1 were significantly lower in KO than in WT livers similar with cultured liver endothelial cells (Figure 7a,b). In addition, heart vessel expression of VE-cadherin was decreased in KO mice as well (Figure 7c). Our observation implies that endothelial CAR is important for stability and localization of the vascular endothelial junction protein and that CAR deletion attenuates endothelial cell junction and increases paracellular permeability.

## 3. Discussion

Our study is the first to address the role of CAR in vascular endothelial cells. Our initial observation was that endothelial vascular permeability was increased by CAR-eKO. When CVB3 was used to infect endothelial cells, it stayed in endothelial cells for a long time and replicated slowly. While CVB3 remained captured by endothelial cells, the innate immune response was activated and protected the heart from CVB3 infection. Thus, we hypothesized that endothelial expression of CAR is very important for inhibition of virus penetration and maintains endothelial para-cellular permeability. In CVB3-induced myocarditis, inflammatory cell infiltration and cardiac fibrosis were not significantly increased in the heart of CAR-eKO mice at 5 days post infection. However, viral titers in the heart, pancreas, and liver were higher in CAR-eKO than in WT mice. In addition, CVB3 infection was observed in the brain of CAR-eKO mice but not in the brain of WT mice. The blood–brain barrier is particularly rich in endothelial tight junctions, and CVB3 are not able to penetrate the blood–brain barrier [25,26,27]. In CAR-eKO mice, vascular permeability was increased by Evans blue dye (EBD) as well as 70-kDa dextran. Moreover, infiltration of inflammatory cells was significantly higher in the peritoneal cavity of CAR-eKO mice than in that of WT mice. Thus, CAR is likely to be involved in molecular mechanisms that regulate endothelial para-cellular permeability. 

CAR is co-localized with adhesion proteins such as β-catenin [14] and cadherin [15]. Additionally, CAR is well known as a co-ligand for JAML protein, which is altered in the immune system with implications for asthma, cancer, and chronic nonhealing wounds [16]. Our observations suggest that CAR is substantially involved in the endothelial adhesion junction stability because CAR deletion in endothelial cells reduced the levels of VE-cadherin and PECAM-1. Cadherins mediate cytoskeletal organization modifications and regulate activity of Rho family GTPases [28]. This signaling activity of VE-cadherins is associated with endothelial junctional strengthening. Furthermore, PECAM-1 regulates endothelial permeability and senses shear stress as a mechanoreceptor complex with VE-cadherin [29,30]. Altogether, our findings show that CAR may be involved in several signaling pathways leading to either endothelial barrier reinforcement or increased permeability.

The present study has several limitations. We could not validate whether CAR directly interacts with endothelial junctional molecules such as VE-cadherin and PECAM. Although all these molecules were co-localized in the endothelial tight junctions, it is still unclear how they communicate with each other. We used isolated hepatic endothelial cells, but each organ endothelial cell has a different character and CVB3 infectivity. There is a limitation that we did not compare different types of endothelial cells. Therefore, it is possible our in vivo findings in the heart and liver may have differences compared to endothelial cells evaluated in vitro. Furthermore, signal pathway regulation was not examined in the present study. We considered the roles of CAR in endothelial cells from the data on endothelial cell junctional protein changes and CVB3-induced myocarditis. Finally, vascular function was not examined in this study. 

In this study, we demonstrated that CAR plays an important role in endothelial cell junctions and in regulation of vascular permeability. End-organ infection with CVB3 is increased in eCAR-KO mice, indicating a role for endothelial CAR in limiting passage of the virus through the vasculature to the underlying tissue. While there was not a significant increase in inflammation or fibrosis at day 5 in eCAR-KO mice, there was an increase in viral titer and Evans blue dye staining, suggesting that endothelial CAR could have an effect on viral heart disease. These data may help to develop novel pharmacological approaches to treatment of coxsackieviral-mediated diseases that affect a variety of organ systems.

## 4. Materials and Methods

### 4.1. Generation of Endothelial-Specific CAR Knockout Mice

A floxed CAR construct was generated by inserting loxP sites just before and after the second exon, as previously described [23]. To generate endothelial-specific CAR knockout (f/f Cre, CAR-eKO) mice, the homozygous floxed CAR mice were bred with Tie2-Cre transgenic mice. Mice were backcrossed with the Balb/C strain of mice more than 5 generations for coxsackievirus infection experiment. CAR-eKO mice were compared to wild-type mice that were homozygous floxed CAR without Tie2-Cre (WT). 

### 4.2. Liver Endothelial Cell Isolation and Culture

Mice were euthanized by 50ug/kg ketamine then livers were collected. Collect livers were moved to a Petri dish and cleaned of fat or excess tissue. Livers were minced using 2 autoclaved razor blades. Minced organs were transferred to a 50 mL tube containing 25 mL collagenase and incubated in 37 °C water bath for 1 h occasionally shaking the mixture. Mixture transferred by pipette through a 70 µm disposable cell strainer into a fresh 50mL tube. Pellet was resuspended in 25 mL of 0.1% BSA and again centrifuged at 1300 rpm for 5 min at 4 °C. Supernatant was aspirated and resuspended again. Resuspended cells were add into a CD31 MACS cell separate system (Miltenyi Biotec, MD) then bead binded cells were isolated following manufacturer’s manual. Cells were incubated by using RPMI1640 medium including 10% fetal bovine serum and 10 ng/mL endothelial growth factor. Primarily isolated liver endothelial cells were not used after 10th passage for experiments. Isolated endothelial cells were used for trans-well chamber experiment for endothelial permeability test. Briefly, isolated WT and CAR-eKO (KO) endothelial cells were cultured in the upper chamber of a trans-well plate (Nunc Co., UK) and the HeLa cells were cultured in a bottom well to define the virus amount that passed the upper-chamber endothelial cell layer.

### 4.3. Determination of Vascular Permeability

In vivo endothelial permeability was assessed with the use of Evans blue dye and 70-kDa dextran-FITC. Briefly, Evans blue dye (EBD, 10 mg/mL) and dextran-FITC (300 ul) was injected through tail vein. After 3 h, heart was collected and snap-frozen by OCT Tissue Tek (Sakura Finetechnical, Torrance, CA, USA). Heart was sectioned by 7 um, then EBD and dextran-FITC uptake levels were observed by fluorescent microscope (Olympus, Waltham, MA, USA) and dye uptake areas were quantified by Scion Image (Scion science, Chicago, IL, USA) density bar tools. For EBD measurement in peritoneal cavity, which were washed by 5 mL PBS, EBD concentration of peritoneal cavity was determined by microplate reader at absorbance at 650nm. Dextran-FITC level was measured by fluorescent microplate reader from the supernatant of heart extracts.

### 4.4. Peritoneal Cavity Immune Cell Infiltration

Peritoneal cavity immune cell infiltration was measured by FACs analysis to compare WT and KO mice. Mice were sacrificed by CO_2_ gas, then 10 mL 1X PBS was injected into peritoneal cavity to wash out immune cells; collected cells were stained by anti-CD11b-PE (Macrophage) and anti-Gr1-FITC (monocyte) antibody (R&D biosystems, Minneapolis, MN, USA). 

### 4.5. Western Blot Analysis

We observed CAR and endothelial cell-to-cell junctional protein expression level; protein was extracted from both WT and KO endothelial cells. Immunoblot analysis used an ECL detection system as described previously [24]. Primary antibodies were used; anti-CAR (Santa Cruz Biotechnologies, Dallas, TX, USA), PECAM-1, VE-cadherin (R&D system, Minneapolis, MN, USA), FLK-1 (Sigma-Aldrich, Inc. St. Louis, MO, USA), integrin-β1D (Zymed Laboratories, Inc. South San Francisco, CA, USA), and GAPDH (Santa Cruz Biotechnologies, Dallas, TX, USA). Protein expression levels were evaluated with Image Lab software (Bio-Rad laboratories, Hercules, CA, USA) based on the signal intensity of each protein using GAPDH as an internal control for protein loading. 

### 4.6. Immunofluorescent Staining

Cultured endothelial cells were fixed with ice-cold butanol for 15 min at room temperature, followed by blocking and permeabilization with 2% BSA and 1% Triton X-100 in PBS and incubated with primary antibodies against CAR (1:100; Santa Cruz, Dallas, TX, USA), VE-cadherin, and PECAM-1 (1:500; R&D system, Minneapolis, MN, USA). The target proteins were visualized with the secondary antibodies conjugated with fluorescence (Alexa fluor 488 and 594, 1:400; Invitrogen, South San Francisco, CA, USA) and Hoechst nuclear stain. Fluorescence images were taken and processed using a fluorescent microscope (Olympus, Waltham, MA, USA) [23].

### 4.7. Coxsackievirus B3 Amplification and Infection of Mice

Coxsackievirus B3 (CVB3) was derived from the infectious cDNA copy of the cardiotrophic CVB3-H3. Virus infected endothelial cells and mouse tissue virus titer were measured by PFU assay as we described previously [24]. Balb/C background CAR-eKO (f/f Cre, KO) and their littermate wild-type (f/f, WT) mice were inoculated by intraperitoneal injection with 2 × 10^3^ plaque-forming units of CVB3 at 8–10 weeks of age. Mice were sacrificed at day 5 and day 8 post-infection and then the heart, liver, spleen, brain, and pancreas were harvested and analyzed. Prior to analysis, mice were injected with Evans blue dye 14 h before being sacrificed. The heart inflammation and myocardium damage were observed by histologic analysis. Hematoxylin and eosin and trichrome staining were performed using 10 um paraffin-embedded sections as described previously [23].

### 4.8. Statistical Analysis

Data are expressed as mean ± SE unless otherwise noted. Statistical significance was evaluated using the unpaired Student’s *t*-test for comparisons between 2 means. For multiple comparisons, one-way analysis of variance (ANOVA) using Tukey–Kramer post hoc test was used. All *p*-values less than 0.05 were considered as significantly different.

## Data Availability

Data is contained within the article.
